# Developmental programming in dogs

**DOI:** 10.1093/biolre/ioag055

**Published:** 2026-07-01

**Authors:** Sylvie Chastant

**Affiliations:** Université Paris-Saclay, UVSQ, INRAE, BREED, F-78350, Jouy-en-Josas, France; Ecole Nationale Vétérinaire d’Alfort, BREED, F-94700, Maisons-Alfort, France

**Keywords:** DoHaD, post-natal growth, low-birth-weight, behaviour, placenta, intra-uterine growth restriction, breed

## Abstract

Developmental programming in dogs, although less studied than in other species, encompasses both nutritional and behavioural influences during gestation and early life. The most critical window corresponds to the first 120 days (pregnancy followed but the first two months of dog’s life), considered the canine equivalent of the human “first 1000 days of life.” Low birth weight, result from the intra-uterine growth, is one of the most documented examples of developmental programming in this species. Its consequences extend long beyond the neonatal period: while early effects include disproportionate head development and increased mortality during the first three weeks, long-term effects include a greater predisposition to overweight in adulthood. Gestational programming is modulated by the dam’s diet, which can affect birth weight, neonatal and paediatric health, and particularly inflammatory status. However, the postnatal period provides opportunities to counteract prenatal influences: the risk of neonatal mortality in low-birth-weight puppies that achieve adequate early growth during the first two days of life becomes similar to that of normal-birth-weight littermates. Conversely, excessive growth during the first weeks of life is suspected to increase the likelihood of adult overweight, highlighting the need for precise management of neonatal and paediatric growth to ensure healthy adult trajectories. Early-life behavioural programming, through maternal behaviour and environmental exposures, is also essential in shaping dogs for their future societal roles. Overall, developmental programming appears to be a key determinant of lifelong physical and mental health in dogs.

## Teaser

Early life shapes dog health and behaviour until adulthood.

## Graphical Abstract

**Graphical Abstract:**
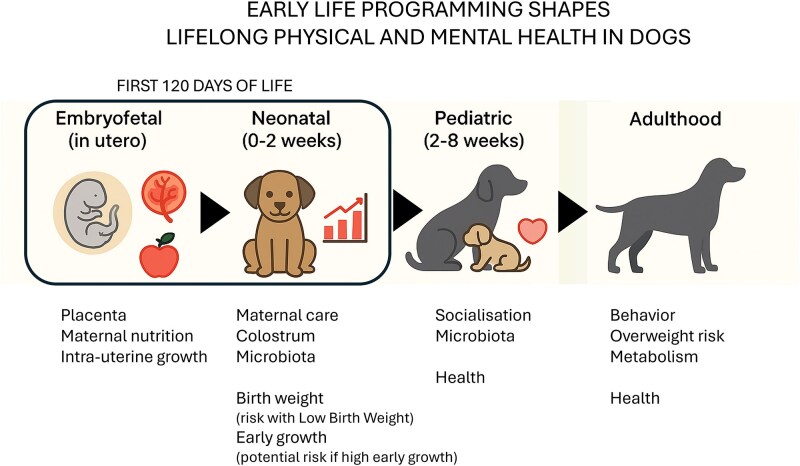

## Introduction

Developmental programming, defined as the influence of events occurring during embryonic, fetal, or neonatal life on offspring health throughout their lifetime, was first conceptualized in humans [1] and has since been demonstrated across a wide range of animal species, from rodents to large domestic animals. Most mechanistic studies have been conducted in mice, rabbits, and sheep, helping to unravel the physiological and molecular bases of the phenomenon [2]. In contrast, knowledge of developmental programming in dogs remains limited. Clinical and phenotypic evidence is still scarce, even though investigating the Developmental Origins of Health and Disease (DOHaD) in this species is of clear interest [3].

To date, research on maternal programming in dogs is still emerging and has mainly focused on the consequences of intra-uterine growth, particularly in relation to low birth weight (LBW). Prenatal nutrition of the dam, as well as postnatal nutrition of both dams and offspring, has received growing attention because of its potential to guide breeder practices and veterinary recommendations. Beyond nutrition, the role of maternal behaviour in shaping puppy temperament has also been explored, given its implications for dam selection.

This review focuses primarily on the embryofetal and early neonatal periods (birth through the first three weeks of life). Nevertheless, programming events can occur later as well, including during weaning (around 6–8 weeks), juvenile growth, and even at neutering. As dogs are increasingly regarded as full family members (“pet parents”), serve in close collaboration with humans in assistance roles, and frequently present behavioural disorders that may strain owner–dog relationships and contribute to shelter overcrowding, the importance of understanding developmental programming in this species is oh high societal importance. Exploring DOHaD in dogs is therefore relevant for both the canine and the human species.

### The dog species

The domestic dog (*Canis lupus familiaris*) lives in close association with humans, whether as a companion animal, a service animal, or a free-roaming stray. In 2023, the canine population was estimated at approximately 106 million in Europe ((https://www.statista.com/) and 90 million in the United States (https://www.avma.org/). Most dogs are kept as pets, with 45.5% of U.S. households and about 30% of French households owning at least one dog (https://www.facco.fr/). The economic value of the European pet market is substantial, reaching an estimated annual 29.1 billion euros for pet food and 24.5 billion euros for related services and products, with continued growth (https://europeanpetfood.org). From a societal and animal-welfare perspective, a notable concern is the annual intake of 3.1 million dogs into U.S. animal shelters, of which approximately 400 000 are euthanized (https://www.aspca.org/).

Zoologically, 373 breeds are recognized within the species, displaying considerable phenotypic diversity, particularly in adult body weight and morphological type (brachycephalic, dolichocephalic, mesocephalic). The dog is the terrestrial mammalian species with the greatest variability in adult size: adult body weight ranges from about 800 g in the Chihuahua breed to nearly 100 kg in the Mastiff (https://www.fci.be/). This remarkable phenotypic diversity reflects a high genetic variability and distinct breed-specific genetic backgrounds [4].

From a breeding standpoint, two main populations coexist. Purebred dogs with documented lineage are bred by individuals who generally maintain a small number of breeding females and operate at low intensity; they represent a minority of the total dog population (around 16% in France, based on identification registries; https://www.i-cad.fr/). Mixed-breed dogs or dogs “of breed type” (i.e. phenotypically similar to recognized breeds but without formal pedigree registration) constitute the majority. These dogs may be bred by private owners or originate from large-scale commercial breeding operations (“puppy mills”), which raise ongoing concerns regarding animal welfare and cross-border puppy trade.

Biologically, the dog is a monoestrous, non-seasonal, polytocous species. Bitches typically exhibit only two estrous cycles per year. Under responsible breeding guidelines, they are bred from approximately 1 year of age until 7–9 years, depending on breed, although no physiological menopause occurs [5]. Average litter size is 5.4 puppies, with substantial breed-related variation and a positive correlation between dam format and litter size [6, 7]. Pregnancy length from ovulation averages 63 ± 2 days [8].

Due to their zonary endotheliochorial placentation, puppies are born nearly agammaglobulinemic and therefore depend on colostrum intake to acquire passive immunity [9]. As an altricial species, newborn puppies are blind, deaf, and display limited motor and cognitive capacities during the first two weeks of life. They depend on maternal stimulation (anogenital licking) for urination and defecation. During the third week, the ear canals open and eyelids separate; puppies begin to urinate and defecate autonomously and progressively develop motor skills [10]. Puppies nurse and remain with their dam until weaning, typically at 6–8 weeks of age.

From the preconception period through pregnancy, lactation, and weaning, dams and puppies are managed within breeding facilities. After adoption, private owners assume responsibility for growth, training, and health management, including decisions regarding neutering [11]. Recent reports indicate a median life expectancy of 11.7–15.4 years, with substantial breed-related variability, ranging from 4.5 to 12.7 years in large population studies [12, 13].

### Potentials of developmental programming in the dog

As the societal role of dogs evolves, interest in preventive medicine has grown. Insights from DOHaD could reshape the management of several conditions by tracing their origins to early life. Obesity, one major consequence of intrauterine growth restriction across species [14], has reached epidemic levels in both humans and dogs. Using a large national database, the Banfield Pet Hospital network estimated that 45%–50% of adult to senior dogs are overweight and 8–13% are obese [15]. This chronic inflammatory disease is associated with serious orthopedic and endocrine comorbidities and significantly impairs quality of life [16, 17].

DOHaD may also help optimize dogs for their intended social roles. Early-life programming could promote traits such as controlled aggressiveness in military dogs, low reactivity and problem-solving skills in guide dogs, or adaptability in companion dogs. Behavioural issues remain a major cause of dog bites and relinquishment [18].

The dog may also serve as a relevant DOHaD model for humans, sometimes more informative than conventional laboratory species. In both species, birth weight strongly influences lifelong health trajectories [19], and both puppies and human preterm infants are born at roughly 2% of maternal weight. Puppies’ neurological immaturity makes them highly sensitive to early environmental influences. At the difference with numerous animal species, this progressive development of complex cognitive abilities further allow assessment of behavioural outcomes [10, 20]. Nonetheless, significant reproductive differences—monotocous hemochorial pregnancy in humans vs. polytocous endotheliochorial pregnancy in dogs—limit direct extrapolation [21]. Dogs may therefore be better viewed as sentinels of human health, sharing lifestyle and environmental exposures with their owners.

In humans, the critical developmental window spans “the first 1000 days,” from conception to two years of age [22]. Using an epigenetic age-equivalence model (human_age = 16 ln(dog_age) + 31) [23], this period corresponds in dogs to roughly “the first 120 days,” encompassing gestation plus the first two postnatal months—the period typically under a breeder’s control. Most canine DOHaD studies currently focus on this earliest postnatal stage, particularly birth weight and the neonatal period (first three weeks).

### Maternal programming through intra-uterine growth: Low birth weight

Intra-uterine growth restriction provided the foundation for the emergence of the DOHaD concept in the human species. As evidenced in other species, low birth weight in the canine species has also been recognized to have short- to long-term consequences (see below). Nevertheless, the exploration of the impact of birth weight in the canine species is made complex by the considerable inter-breed variability of this parameter. Weight differences at adulthood are already apparent from birth, with Chihuahuas being born at a median weight of 118 g vs 640 g for Newfoundland puppies (breeds of respective adult weight expected at 800 g for a Chihuahua vs 68 kg for Newfoundlands) [24, 25]. This variability requires analysis to be conducted at the breed level rather than at the species level.

Based on significantly increased risks of neonatal mortality, birth weight thresholds defining LBW and even VLBW (Very Low Birth Weight) were calculated for 12 different breeds [26]. In comparison with normal birth weight puppies, whose neonatal mortality rate is 4%, low birth weight puppies are at an increased risk of 11%, whereas very low birth weight puppies face a dramatically higher risk of 61%, based on 4971 puppies from 10 breeds [26]. Age at death also differs according to birth weight: Low and Very Low BW puppies die earlier than others, with a significantly higher proportion of death cases during the first two days of life (48% of neonatal deaths vs 35%) [27].

Interestingly, the sensitivity to birth weight restriction differs between breeds. Rottweiler and Labrador puppies are born at very similar weights (404 ± 59 g vs 410 ± 70 g) in litters of similar sizes (7.6 ± 2.0 vs 7.3 ± 2.6 total puppies per litter) and reach similar adult weights. Nevertheless, the thresholds defining VLBW as calculated by CART analysis markedly differ between the two breeds: respectively 345 g and 248 g. This suggests that Rottweiler puppies suffer from complications of intra-uterine growth restriction (IUGR) at a lower growth restriction than Labradors, i.e. a higher susceptibility of Rottweiler puppies to reduced intra-uterine growth. Prevalence of VLBW puppies also exhibits large variations between breeds: 13.5% in Rottweilers vs only 1.4% in Labradors [26]. Metabolic and (epi)genetic mechanisms underlying this susceptibility remain to be explored.

Despite being clearly distinct from heavier-born puppies, it remains to be determined whether LBW corresponds to small for gestational age (SGA) or intra-uterine growth restricted (IUGR) puppies, the first ones being small but otherwise healthy, whereas the second display pathological traits and higher risk of mortality and morbidity. IUGR piglets differ from constitutionally small ones by a steep, dolphin-like forehead, bulging eyes, and wrinkles perpendicular to the mouth [28, 29]. Developing similar morphological criteria in dogs could help identify fetuses affected by IUGR, potentially leading to severe long-term developmental and health consequences. Antenatal Doppler evaluation of uteroplacental function, used to detect IUGR in humans, is difficult to implement in dogs due to the presence of multiple fetuses in the uterus [30, 31]. Moreover, weight thresholds defining LBW were calculated based on the risk of neonatal mortality: it remains to be determined whether they are also relevant for predicting long-term adult outcomes.

### Neonatal consequences of LBW

**Figure 1:**
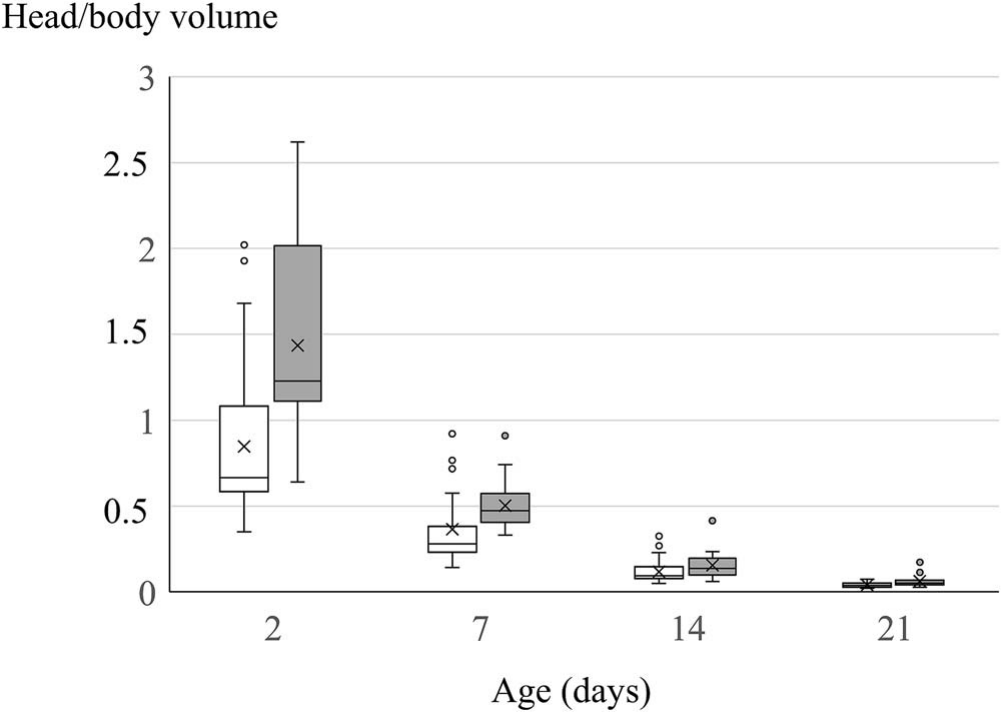
Head/body volume in normal (white; n = 34) and in low-birth-weight (grey; n = 16) puppies over the first three weeks of life. Head volume is calculated from the skull circumference, considering the head as a sphere. Body volume is calculated from the thoracic perimeter and the occiput-rump length, considering the body as an ellipse. ^***^P < 0.001; ^**^P < 0.01

In the post-natal period, maternal intra-uterine programming impacts puppy anatomy and physiology over the neonatal period. At birth, LBW puppies are morphologically different from normal birth weight puppies (NBW), with a higher head/body volume ratio; this relative overdevelopment of the skull persists over the first three weeks of age (fig. 1). In sheep and human fetuses subjected to growth restriction, responses to chronic hypoxia include the redistribution of cardiac output to favor essential organs, including the brain [32]. Such hemodynamic adaptation, termed “brain sparing,” increases the brain-to-abdomen ratio, reflecting relative brain overdevelopment. Morphology of LBW puppies suggests that this brain-sparing effect may also exist in the canine species. Nevertheless, in IUGR infants, brain sparing does not ensure fully normal cerebral development; affected babies suffer from structural abnormalities (such as decreased cortical development, deficits in neuronal myelination and connectivity) and functional impairments including deficits in motor skills, cognition, memory, and neuropsychological disorders such as schizophrenia, depression, anxiety, and autism [32–35]. Consequences on cognitive and mental competencies during the paediatric period and adulthood remain to be explored in dogs.

During the first 48 h of life, a crucial step for survival and growth is colostrum ingestion, providing passive immune transfer (when ingested during the first 12 h of life, i.e. until the closure of the intestinal barrier) and energy. In LBW puppies, the number of colostrum suckling sessions and the total duration of suckling over the first 12 h of life are lower than in NBW puppies [36]. This suggests the ingestion of a lower quantity of colostrum, confirmed by a lower growth rate of LBW puppies over the first 2 days of life [27]. In addition to growth, this reduced colostrum intake may also affect microbial seeding of the digestive tract. As soon as 2 days of life and over the first three weeks, the digestive microbiota of LBW puppies exhibits major shifts compared to heavier-born puppies [37]. Three bacterial families were found in higher relative abundance in the gut of LBW puppies: Enterobacteriaceae (mostly *E. coli*), Clostridiaceae (mostly *Clostridium* genus), and Lachnospiraceae (including the genus *Tyzzerella*). These families are opportunistic and can create conditions favoring disease, including inflammatory bowel disease in dogs [38]. Indeed, neonatal deaths are preceded by digestive symptoms in 54% of puppies [39]. Garrigues et al [37] hypothesized that reduced colostrum ingestion may be associated with a lack of strict aerobic bacteria in the gut of LBW puppies, preventing adequate oxygen consumption in the gastrointestinal tract during the first days of life. The resulting higher oxygen levels may induce earlier and increased colonization by facultative anaerobes, along with delayed colonization by strict anaerobes. In preterm infants, similar deviations in digestive microbiota are observed and are known to alter the intestinal barrier and host immune function [40].

Altogether, lower energy intake, higher risk of passive immune transfer deficit, and altered gut microbiota in LBW puppies contribute to the higher risk of neonatal and even paediatric (up to 2 months of age) mortality.

### Consequences of low birth weight in adulthood 

**Figure 2:**
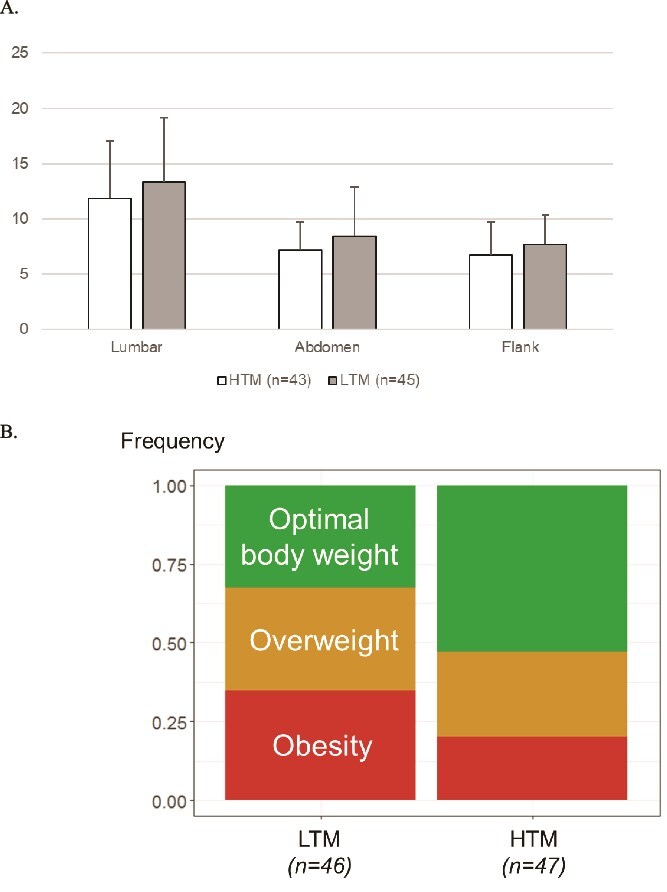
Relationship between body composition at adulthood and birth weight. LTM: Lighter than median at birth; HTM: Heavier than median at birth. A. Subcutaneous fat thickness. Individuals with the lightest birth weights had thicker fat deposits whatever the site of measurement (lumbar, abdomen, flank; *P* = 0.014; n = 88 Labradors) [43] B. Proportion of overweight and obese adults depending on birth weight (*P* = 0.032; n = 93 Labradors) [42].

In LBW puppies that survive the neonatal period, the anatomical and microbiota differences observed early in life progressively diminish and are no longer detectable by one month of age (Figure 1) [37]. However, consistent with findings in humans and other species [41], adult dogs born LBW exhibit altered body composition. In a cohort of Labrador Retrievers managed under strictly standardized breeding and early-life conditions, the prevalence of overweight and obesity in adulthood was significantly higher in LBW dogs compared with NBW controls (70% vs. 47%) [42]. Beyond body weight and Body Condition Scores (BCS), ultrasonographic assessments of subcutaneous fat thickness further confirmed altered adiposity in adults born LBW (Figure 2) [43].

The increased obesity risk in LBW dogs may reflect the development of a “thrifty phenotype” during the fetal life, whereby nutrient restriction in utero induces metabolic adaptations favouring postnatal energy conservation and fat storage [28, 44]. Differences in early gut microbiota colonization—previously shown to vary between lean and obese dogs [17] and to differ in LBW puppies—may represent an additional pathway contributing to obesity in adulthood.

### Maternal gestational programming: onset of deciphering

Very few aspects of the so-called “maternal environment” responsible for embryofetal programming have been explored in the canine species: placental development, position within the uterine cavity, sex of the surrounding fetuses, and, as classically in a DOHaD approach, maternal nutrition during pregnancy.

**Figure 3:**
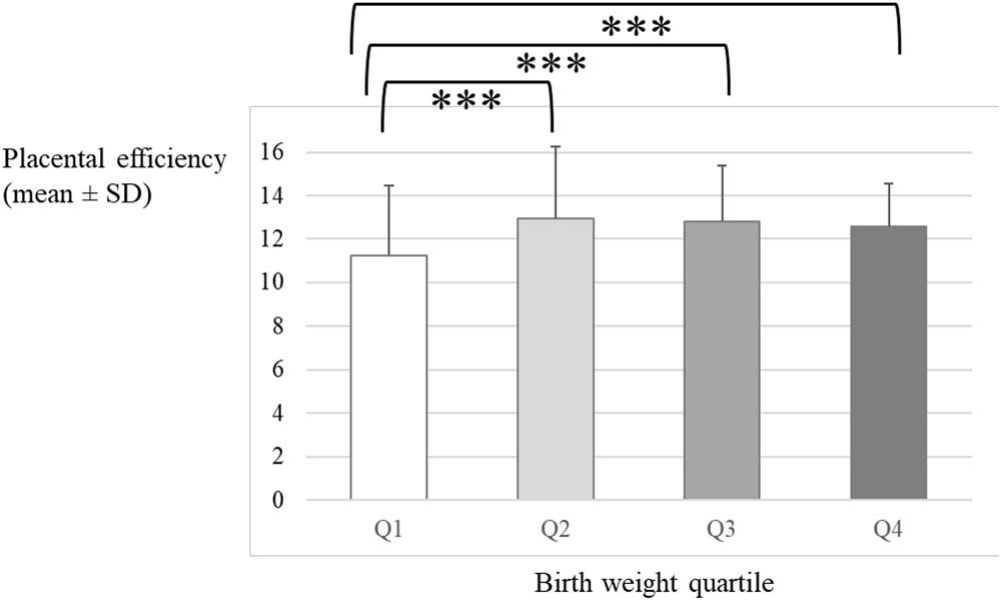
Placental efficiency depending on birth weight (n = 169 Boerboel puppies). Placental efficiency was calculated as the ratio weight of the placental attachment zone/birth weight. For birth weight, Q1 are the 25% lightest puppies at birth. ^***^P < 0.001

The influence of placental development and intra-uterine position on puppy birth weight can be ethically studied via the extraction of fetoplacental units during term C-sections. Birth weight appears unaffected by gross or histological abnormalities [45], only weakly correlated with placental vascular density, and moderately correlated with placental weight or surface area (r^2^ = 0.38 for both; n = 187 fetoplacental units, author’s unpublished data) [46]. Nevertheless, placental efficiency, defined as the ratio of birth weight to placental weight, is significantly lower in LBW puppies (see puppies of the lowest quartile Q1 in Figure 3), suggesting that placental development may be a limiting factor of fetal growth.

Intrauterine position could also influence fetal growth in polytocous species, by affecting both space availability for conceptus development and nutrient supply to fetuses. In dogs, the uterus receives blood supply through anastomoses of the ovarian and uterine arteries, whose respective contributions are controversial [47]. Regardless of the predominant artery, a nutrient gradient is established along the uterus. In a cohort of 19 bitches, while the heaviest puppy of the litter was found at the apex of a uterine horn in 11/19, no preferential position within the genital tract (apex/horn/base of the horn/uterine body) was observed for the lightest puppy, suggesting that nutrient provision is not the limiting factor of LBW intra-uterine growth (author’s unpublished results). One could have hypothesized that apex development might be limited by the blind ovarian extremity, but this was not supported by observations in this cohort. This result reinforces the hypothesis of predominant vascularization via the ovarian artery, with the fetoplacental unit at the apex receiving the highest nutrient concentration. No increased risk of neonatal mortality has been evidenced in the high birth weight puppies. Nevertheless, long-term consequences of excessive fetal growth would be interesting to consider in dogs, since in humans Large for Gestational Age infants suffer at adulthood from diseases similar to those described in LBW babies [41]. During fetal development, each conceptus is in close contact with one or two fetuses, allowing biochemical exchanges, including androgen transfer. Unlike some other species [48], no impact of surrounding fetuses’ sex on birth weight has been evidenced in dogs, but its influence on adult behavior, as seen in rats [49], remains to be evaluated, considering the importance of behavior in dog–human interactions.

Bitch metabolism and nutrition also contribute to the programming of offspring neonatal and long-term health. Maternal body condition affects LBW prevalence. The proportion of LBW puppies is significantly lower in lean or ideal BCS bitches compared to overweight or obese females at mating (15.5% vs 26.0%) or mid-pregnancy (11.3% vs 31.5%) [50]. In addition to absolute Body Condition Score, the impact of its variation over pregnancy warrants further investigation. Due to the high prevalence of excessive adiposity in breeding bitches (59%; [50]), special attention should be paid to BCS control long before breeding. The programming effects of maternal nutrition during pregnancy have been evidenced at birth, during the pediatric period, and at adulthood. Supplementation with *Saccharomyces cerevisiae* var. *boulardii* from Day 30 of pregnancy until whelping was associated with a decreased proportion of LBW puppies: 13% with yeast vs 29% without supplementation (OR: 3.5) [51]. Supplementation during the second month with a combination of prebiotics (mannan- and fructo-oligosaccharides) and probiotics (*Enterococcus faecium* and *Lactobacillus acidophilus*) was associated with a dramatic decrease in digestive disease episodes in puppies during the first 9 weeks of life [48, 49]. Maternal diet also programs offspring inflammatory status: when dams’ diet is dominated by ultraprocessed carbohydrate-based food (kibbles), odds ratio for otitis in their puppies during the first year is 1.6 (95% CI: 1.14–2.40) compared to a non-processed meat-based diet ([52]; n = 3064 dogs).

### Early life programming: nutritional

**Figure 4:**
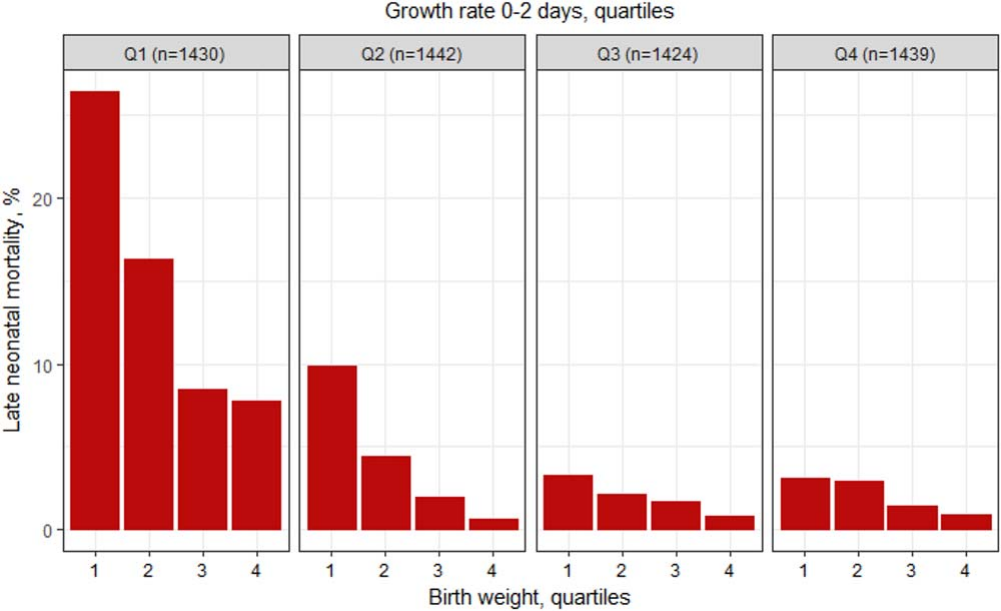
Modification of maternal programming by early growth (0–2 days). N = 5735 puppies from 12 differents breeds. Birth weight quartiles were defined within each breed. Within each birth weight quartile, puppies were then classified depending on their growth rate over the two first days of life (calculated in % of their birth weight). Mortality was recorded from Day 2 to Day 21 of life.

Not only the fetal life, but also post-natal events program the health trajectory of a dog. As early as the first 2 days of life, neonatal programming can reverse some of the effects of gestational programming. Ensuring a high growth rate over the first 2 days of life in LBW puppies dramatically decreases the Day 2–60 mortality rate to levels close to those observed in NBW puppies (Figure 4) [27]. The objective is a growth rate higher than −1.8% over the first day and −0.4% over the first two days of life, sufficient to mitigate the risk of mortality over the first 2 months. In practice, breeders are advised to feed puppies so that they maintain birth weight over the first two days.

Colostrum is thus of major importance, both quantitatively and qualitatively. Nutritional colostral quality can be modified by maternal diet during pregnancy. Probiotic supplementation with *S. cerevisiae* var. *boulardii* during the second month of pregnancy increased colostral gross energy (+12%) via a 17% increase in sugar concentration. Results of pre/probiotic supplementation on immune quality of colostrum are controversial, with either an increase [53, 54] or no effect [51] on IgG colostral concentration.

Growth from Day 2 to Day 7, i.e. relying on milk, also reverses the effects of LBW [27]. “Lactogenic” programming, for example by extending maternal supplementation with pre/probiotics after whelping, impacts puppies’ inflammatory status at 56 days of life, with a markedly decreased IL8/IL10 ratio in blood, indicative of better control of systemic inflammation [51]. Conversely, a diet demonstrated as proinflammatory during pregnancy has no effect when ingested by dams during lactation [52].

If early-life growth rates must exceed determined thresholds to improve survival, optimal growth may not be maximal growth, and post-natal programming via growth can also be detrimental for adult health. Specifically, high growth rates in early life are suspected to increase risk of obesity in adulthood [55]. According to Leclerc et al. [56], studying a neonatal–paediatric cohort of female Beagles neutered at 8 months, a growth rate over the first 2 weeks >125% was associated with a higher proportion of obese animals at 2 years. Conversely, in a mixed-sex Labrador cohort (neutered or intact), no critical threshold for early growth over the first 2 days or 2 weeks could be determined regarding overweight risk [43]. Interaction between neutering and neonatal programming warrants further study.

In addition to quantitative growth (colostrum/milk intake), the quality of early-life diet is also critical. Since composition of industrial milks differs from canine colostrum/milk, particularly in energetic value, fatty acid profile, amino acids, calcium and phosphorus concentrations [57, 58], it would be interesting to explore whether artificial feeding programs individuals differently than maternal colostrum and milk.

Not only neonatal but also paediatric growth programs adult body condition. In an epidemiological study of >200 000 privately owned dogs, Salt et al. [59] demonstrated that 83% of dogs overweight by early adulthood (3 years) had a growth rate between 12–60 weeks higher than predicted by their growth chart. The same was true for 67% of dogs with diseases associated with accelerated growth (hypertrophic osteodystrophy, hip dysplasia, osteochondritis dissecans). Similarly, Montoya et al. [15] showed that a vast majority of dogs overweight or obese during growth remain so at adulthood (67% for overweight ones, 75% for obese), highlighting the importance of early-life programming for health trajectory.

### Early life programming: behavioural

The influence of early life on social development in dogs is an active area of research. It is especially important in dogs, for which specific behaviours are expected (e.g., assistance dogs) and others considered inappropriate. Behavioural disorders, such as aggression towards dogs or humans, anxiety (barking, destruction), are major causes of relinquishment, sheltering, or euthanasia [18, 60]. According to WHO, dog bites cause tens of millions of injuries annually, approximately 4.5 million in the United States alone (https://www.who.int/news-room/fact-sheets/detail/animal-bites). A recent study involving 300 dogs reported that 57% displayed at least one owner-perceived undesirable behaviour [61], underscoring the societal importance of early-life programming of socio-emotional traits.

Although much of canine neurological development occurs *in utero*, maturation continues until at least 36 weeks of age. This prolonged postnatal period includes changes in brain electrical activity, dendritic growth, synapse formation and stabilization, and progressive myelination [20]. Behavioural and cognitive traits are therefore highly sensitive to environmental influences across several developmental phases: gestation, the neonatal period (0–2 weeks), the transitional phase (2–3 weeks), early socialization (≈3–12 weeks), late socialization (12 weeks to ~6 months), and adolescence, extending to around one year of age [10, 20]. Experiences during each phase exert cumulative effects on trainability, health, and performance [10, 62].

From birth onward, puppies receive essential behavioural inputs from their dam. Maternal care extends beyond milk provision and includes oronasal interactions, licking, nursing posture, and exposure to social and environmental cues [63]. As the primary adult model, the dam plays a central role in shaping puppy behaviour. Puppies separated from the mother at 30–40 days—thereby deprived of maternal care during the second month—show increased fearfulness, noise reactivity, and excessive barking compared with those separated at 60 days [64]. Low maternal interest, as perceived by owners, is likewise associated with more fearful behaviours in adulthood [65].

Depending on the intended role of the puppy, specific temperament traits are desirable and can be influenced by maternal programming (e.g., selecting dams with high or low maternal investment) or by early environmental enrichment. Military and police dogs require high search focus, sharpness, prey drive, aggression, and engagement. Drug-detection dogs must show obedience, sustained activity, and concentration. Guide dogs require obedience, problem-solving skills, adaptability, and low reactivity or anxiety.

Maternal care can be quantified during the first 3 weeks postpartum using indices that integrate time spent with the litter, physical contact, nursing behaviour, licking, and investigative interactions. Puppies are then evaluated at 12–15 months for performance outcomes. High maternal care may be beneficial or detrimental depending on the dog’s intended purpose. In German Shepherds bred for military use, litters raised by highly attentive dams showed higher scores for social engagement, physical engagement, and aggression, all traits aligned with their future tasks [66]. Conversely, in guide-dog programs, puppies from dams with high maternal investment had a lower success rate, with an odds ratio of 4.62 for release from training. High maternal care was associated with reduced cognitive performance and increased anxiety [67, 68]. It has been hypothesized that puppies exposed to lower levels of maternal nursing (including more demanding suckling postures such as vertical nursing) may develop adaptive strategies when confronted with early-life challenges, resulting in better problem-solving and persistence as young adults. By contrast, puppies receiving intensive maternal care face fewer early challenges and later show slower task resolution and reduced perseverance [67].

Not only maternal behaviour, but also environmental stimulations contribute to behavioural programming. Comparisons between puppies raised in home environments [68] and those from laboratory colonies [37] at two months of age revealed that extensive maternal care in home settings was associated with greater separation distress, whereas laboratory-raised puppies showed more environmental engagement and less destructive behaviour, likely reflecting differences in early stimulation. Additional early-life stimuli, such as radio broadcasts during the first month, have been shown to produce more appropriate behavioural responses at 7 weeks [69]. Exposure to diverse environmental stimuli from 4 to 14 weeks is critical for socialization and successful integration into human society.

Distinguishing genetic from environmental influences remains challenging because dams rear their own genetically related offspring. To isolate the specific effects of maternal behaviour, cross-fostering half-litters between dams with contrasting care styles would be required.

### Establishing dog health trajectories

The embryofetal period and the first two months of life have been demonstrated as critical for developmental programming. Since these periods are supervised by breeders before adoption, defining and disseminating guidelines for breeding, pregnancy, and early-life management is of high importance. Not only veterinarians but primarily breeders must become aware of their role in developmental programming to give puppies the best chances in life. Despite evidence that LBW is a major risk factor for neonatal and paediatric mortality, a large majority (~75%) of breeders are not aware of this short-term influence (69) on 649 breeders). Most are probably also unaware of the long-term consequences of LBW and suspicions about elevated early growth rates. Knowledge of DOHaD in dogs should encourage precise monitoring of early growth (i.e. sufficient to limit mortality but not excessive to avoid overweight in adulthood) and careful control of artificial feeding during the first days of life. Regular weighing of puppies from birth through the first two months should be encouraged as an easy and low-cost health monitoring tool. Reference “healthy” growth curves are now available for puppies, similar to those established for human infants [70]. Moreover, puppies born at LBW and/or exhibiting excessive early growth require careful lifelong monitoring of body condition and nutrition. Their neonatal and paediatric growth data should be shared by breeders with adopters to guide long-term care.

This underscores the importance of health records that integrate neonatal, paediatric, and adult health data. Continuity is especially challenging in the canine species, where puppies are born and raised in kennels for the first two months and then individually adopted. Prospective lifelong cohort studies are hindered by this discontinuity between breeders and owners, as well as by the dispersed nature of pet ownership. Unlike many other animal species, there is no coherent network for long-term follow-up of canine health. Data collection is further complicated by the small number of puppies per breeding facility, the fact that male and female breeding dogs are often owned by different people, and the large number of pet parents (each usually adopting only one dog). These factors make it difficult to establish relationships between early-life (including embryofetal) exposures and adult health, in addition to ethical limitations on exploring the embryofetal period.

Whatever “whole life” datasets covering birth (and ideally preconceptional parental data) and the entire lifespan (up to ~15 years) would be key for DOHaD exploration in dogs. Laboratory colonies have limited value for such life-course epidemiology due to biased environmental exposure. Databases from veterinary clinics often lack data before 3 months of age but can include millions of animals [15]. Critical points for the clinical and scientific value of canine cohorts include selection of parameters to record (among many environmental exposures and clinical data) and decisions about standardizing life conditions (e.g., diet), breed (single vs. multiple breeds), or neuter status. Sensitivity windows may vary between breed formats and even among breeds within a size category due to differences in growth and maturation kinetics. Moreover specific genetic susceptibilities to DOHaD remain to be explored. Specific genetic backgrounds, such as Labradors or Flat-Coated Retrievers with a 14-base pair deletion in the *POMC* gene associated with food motivation and obesity [71], may influence developmental programming, including intrauterine and postnatal nutrient provision or neutering. Neutering is common in dogs, with 30%–70% undergoing the procedure, depending on country. Performed surgically, neutering can occur during the paediatric period (“early neutering,” <16 weeks) or early adulthood (~puberty). Gonad removal causes major hormonal changes (e.g., steroid suppression, increased LH), significantly impacting later health depending on breed, sex, and age at neutering. In addition to the long-known increased risk of overweight and urinary incontinence, epidemiological data have accumulated over the last 10 years to evidence neutering as a risk factor for tumoral, orthopaedic, immune and behavioural diseases [72, 73]. Beyond its role in postnatal programming, neutering may interact with developmental programming during gestation and the neonatal period, potentially revealing or masking the effects of early-life events. A critical question for dog health, veterinary practice, breeders, and owners is the interaction between neutering and LBW or early growth in the first 2 months: is the risk of post-neutering overweight increased in LBW or rapidly growing puppies compared to normal birth weight counterparts?

## Conclusion

Developmental programming is important not only for a dog’s health trajectory but also for the quality of its relationship with humans. DOHaD, including potential transgenerational effects not yet studied in dogs, opens new opportunities to improve canine health, particularly for obesity prevention, now recognized as a population-level disease. It calls for life-course epidemiology based on “big data” from large, phenotypically characterized populations, involving all stakeholders—breeders, owners, veterinarians, laboratories, and nutritionists. For both physical and mental health, DOHaD paves the way for personalized medicine and nutrition, with medical follow-up and diets potentially tailored to developmental programming (e.g., birth weight and early growth). Canine DOHaD should also be considered from a One Health perspective, as dogs share many environmental exposures with humans.
